# Editorial: Functional and Structural Brain Alterations in Headache: A Trait or a State?

**DOI:** 10.3389/fneur.2020.00859

**Published:** 2020-08-12

**Authors:** Roberta Messina, Peter J. Goadsby, Massimo Filippi

**Affiliations:** ^1^Neuroimaging Research Unit, Division of Neuroscience, Institute of Experimental Neurology, Milan, Italy; ^2^Neurology Unit, Milan, Italy; ^3^NIHR-Wellcome Trust King's Clinical Research Facility, King's College London, London, United Kingdom; ^4^Neurophysiology Unit, IRCCS San Raffaele Scientific Institute, Milan, Italy; ^5^Vita-Salute San Raffaele University, Milan, Italy

**Keywords:** headache, neuroimaging, migraine, biomarkers, secondary headaches

Headache disorders affect billions of people and are among the most disabling diseases worldwide (1). The recognition of the significant economic and social impacts of these conditions has increased the interest in understanding their pathophysiology and has led to the development of new treatments. Neuroimaging studies have shed light on the mechanisms of primary and secondary headache disorders, exploring the structure and function of brain regions that mediate the pain and other symptoms present during the headache attacks (2). However, the biology underlying neuroimaging findings in headache patients is not fully understood. Some of the brain alterations might represent a trait that predispose to the development of headache. Others might constitute an adaptive response to the recurrence of headache attacks. This Research Topic aims to outline the current state of the art on functional and structural brain abnormalities in headache disorders, and highlight different opinions and perspectives whether these brain changes might represent headache brain traits, states or a combination of both. Thirteen papers were published as part of the Research Topic covering different forms of primary and secondary headaches.

It is not surprising that most of the articles in the Research Topic were focused on migraine, being the most frequently studied form of headache. Migraine is now widely accepted as a complex neurological disorder characterized by headache and a plethora of sensory, cognitive and neurovegetative symptoms. Many migraine patients experience non-headache symptoms many hours before the onset of the migraine pain, during the premonitory phase of the migraine attack. Karsan and Goadsby reviewed recent functional imaging studies that have attempted to image migraine patients during this early phase. It emerged from the review that changes in the functional connection between the brainstem, hypothalamus, limbic, and pain modulatory regions may account for the symptoms that patients report during the premonitory phase. The authors hypothesized an early activation of subcortical and diencephalic brain areas, which then exert a top-down effect on brainstem regions involved in trigeminovascular nociception, leading ultimately to headache, and associated symptoms.

Between 15–30% of migraine patients experience aura. Migraine aura symptoms can be very heterogenous between patients and among migraine attacks in the same patient. Using a scoring system that evaluates the complexity of aura symptoms, Petrusic et al. have shown that migraine patients with aura can be stratified into different clinical phenotypes. More complex aura symptoms were associated to a thicker visual and somatosensory cortex. Whether migraine with and without aura are two different entities is still a matter of debate. Faragò et al. and Kincses et al. examined functional and structural brain differences between migraine patients with and without aura, suggesting that these two subtypes of migraine should be handled separately in future studies.

The role of the visual network in migraine pathophysiology is well-known. Puledda et al. reviewed the most relevant neuroimaging studies highlighting the involvement of the visual pathways in the broader spectrum of migrainous disorders, including migraine with and without aura, and in the visual snow syndrome, which is not a form of aura. The visual snow syndrome is a recently recognized neurological disorder characterized by the constant vision of tiny flickering dots covering the entire visual field. In the review, the authors hypothesized that a combination of altered peripheral visual stimulation, thalamic and visual cortical dysfunctions could account for the visual illusion experienced by patients with visual snow.

Mechanisms responsible for migraine chronification are still unclear. Two studies of the Research Topic (Filippi and Messina; Chen et al.) highlighted recent imaging studies exploring the function and structure of brain areas that could have a role in migraine evolution into a chronic form.

Another interesting topic that has been discussed by Maleki and Androulakis is the presence of sex-related differences in migraine patients. So far, only few neuroimaging studies have compared female and male patients with migraine showing distinct structural and functional alterations in pain processing brain areas. These preliminary findings may pave the way to further studies that could explain the higher prevalence of migraine in women.

Similar to migraine, the use of advanced imaging techniques have yielded new insights into the pathophysiology of cluster headache. Over the last decades, an increasing number of neuroimaging studies, reviewed by Ferraro et al., have revealed an abnormal activation and morphology of brain regions, including the hypothalamus and midbrain tegmentum, which can explain the typical circadian and circannual rhythms of cluster headache attacks, as well as the predominant presence of autonomic symptoms.

Abnormalities in the structure and function of brain areas involved in the processing of the cognitive, affective and sensory aspects of pain have also been revealed in patients with secondary headaches. Thus, raising the question whether it is possible to identify a specific imaging pattern for each different headache phenotype. The reviews by Schwedt and Chong highlighted imaging similarities and differences between migraine and secondary headaches, like medication overuse and post-traumatic headache. Although, there is evidence showing that patients with post-traumatic headache experience distinct brain alterations compared to patients with migraine, further research is necessary to define better the alterations directly attributable to the underlying brain injury, post-traumatic headache or a possible pre-injury migraine. Patients with medication overuse headache showed brain changes not only in regions that are part of the pain network but also in areas implicated in addiction. Interestingly, some of these abnormalities normalized following discontinuation of the overused medication, while others persisted. These findings suggest that some brain alterations are secondary to the frequent intake of acute treatments and associated headache, whereas others might represent a brain trait that predispose to development of medication overuse.

Although headache diagnosis is mainly based on taking a good clinical history, diagnostic tests, including brain imaging and imaging of the retina, have a pivotal role in the diagnostic work up of secondary headaches, such as headache attributed to idiopathic intracranial hypertension, as suggested by Moreno-Ajona et al.

In conclusion, the articles included in this Research Topic provided an overview on the main neuroimaging findings in primary and secondary headache disorders. It stands out from the Research Topic that headache disorders are complex neurological conditions. Headache patients experience widespread brain functional and structural alterations, some of which can predispose to a specific headache phenotype, while others can be associated to the perception of headache pain and can change dynamically over time. In the future, novel advanced neuroimaging techniques and computational methods, as machine learning approaches (Messina and Filippi), can improve our understanding of the underlying biology of headaches, leading to the development of novel headache-specific treatments.

## Author Contributions

RM wrote the first draft of the manuscript. PG and MF edited the final version of the manuscript. All authors contributed to the article and approved the submitted version.

## Conflict of Interest

The authors declare that the research was conducted in the absence of any commercial or financial relationships that could be construed as a potential conflict of interest.
